# Emerging nanomedical strategies for direct targeting of pediatric and adult diffuse gliomas

**DOI:** 10.1038/s41416-022-01884-6

**Published:** 2022-06-17

**Authors:** Christy Kolsteeg, Esther Hulleman, John Bianco

**Affiliations:** 1 University Medical Center Utrecht, Heidelberglaan 100, 3584 CX Utrecht, The Netherlands; 2 Princess Máxima Center for Pediatric Oncology, Heidelberglaan 25, 3584 CS Utrecht, The Netherlands

**Keywords:** CNS cancer, Targeted therapies

## Abstract

High-grade gliomas, in particularly diffuse midline glioma, H3K27-altered in children and glioblastoma in adults, are the most lethal brain tumour with a dismal prognosis. Developments in modern medicine are constantly being applied in the search for a cure, although finding the right strategy remains elusive. Circumventing the blood–brain barrier is one of the biggest challenges when it comes to treating brain tumours. The cat and mouse game of finding the Trojan horse to traverse this barrier and deliver therapeutics to the brain has been a long and hard-fought struggle. Research is ongoing to find new and feasible ways to reach specific targets in the brain, with a special focus on inoperable or recurring brain tumours. Many options and combinations of options have been tested to date and continue to be so in the search to find the most effective and least toxic treatment paradigm. Although improvements are often small and slow, some of these strategies have already shown promise, shining a light of hope that finding the cure is feasible. In this review, we discuss recent findings that elucidate promising but atypical strategies for targeting gliomas and the implications that this work has on developing new treatment regimens.

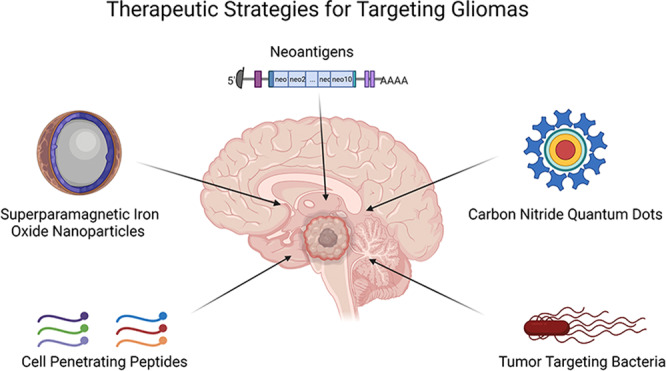

## Introduction

High-grade gliomas are the most lethal primary malignancy in the central nervous system (CNS), both in children and adults, due to their localisation and invasive growth. Gliomas represent 80% of all malignant primary brain tumours and are responsible for most deaths from these tumours [1]. Glioma management is often complicated by an inoperable localisation in the brain, minimising or excluding surgery as a feasible treatment option. In children, high-grade gliomas are typically localised in the supratentorial region or within the brainstem, in which case they are rendered unresectable [2].

**Fig. 1:**
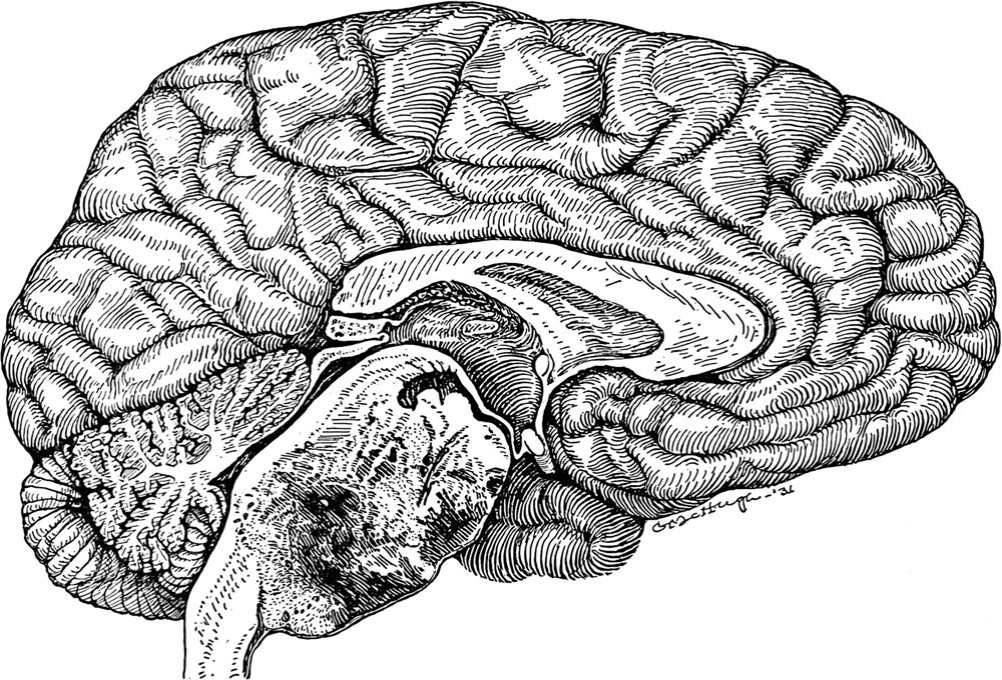
A schematic of a brain section containing a tumour in the pontine region. Adapted with permission from ref. [4]. Median section of brain with spongioblastoma of the pons.

The term glioma was coined over 150 years ago, and since that time a variety of gliomas have subsequently been described. Bassoe and Apfelbach first described diffuse intrinsic pontine glioma (DIPG) in two cases, a girl, and a boy, both aged 6 years old, in 1925, detailing its infiltrative and destructive nature [3]. Two cases of glioma of the pons in children were also described by Bailey in 1933 and showed the realisation that these tumours were not operable (Fig. 1), and that prognosis was poor, with therapy being often restricted to radiation alone [4]. At the time, gliomas in children were thought to be identical to gliomas in adults, and thus treated in the same way, with little success. For adult gliomas, it was not until 1926 when Harvey Cushing and Percival Bailey published their classification of the tumours of the glioma group that glioblastoma (GBM) was distinguished as a distinct entity from other glioma types [5]. Unlike GBM, it has taken nearly a century of research for DIPG to be distinguished as a distinct type within the latest edition of the WHO Classification of Tumours of the Central Nervous System (WHO CNS5), now being termed “diffuse midline glioma, H3K27-altered” [6]. Many classifications describing the different glioma subtypes have been published over the last century, with more than 120 types of brain and CNS tumours being categorised today, from benign to aggressive, with some being assigned a grading system that aids to predict growth and behaviour, and ultimately prognosis. The more recent emergence of powerful molecular profiling and bioinformatic techniques have allowed us to accurately identify molecular differences between CNS tumours such as medulloblastoma, ependymoma, astrocytoma, glioblastoma and diffuse glioma and as such guide treatment decisions by adjusting the therapeutic strategy based on the current molecular profile of the tumour. To this end, WHO CNS5 incorporates histology with molecular data in classifying CNS tumours, illustrating the molecular heterogeneity of the various distinct types with different histopathological characteristics and molecular profiles, resulting in an accurate reclassification of CNS neoplasms [6, 7].

To date, treatment for high-grade gliomas remains inadequate, with 95% of patients succumbing to their disease within 5 years after diagnosis [8]. Standard therapeutic regimens current today usually involve a three-pronged approach consisting of maximal safe surgical resection (when possible), followed by radiation and chemotherapy. Together, these commonly prolong survival by only months. Other causes for therapeutic failure include the diffuse invasion of glioma cells into the surrounding brain, often sheltering them from both surgery and radiation, and the challenge of delivering sufficient doses of chemotherapy across the blood–brain barrier (BBB) [1].

Considering the overall poor outcome from current treatment options for both operable and inoperable glioma, the need for improved targeted therapies becomes evident in overcoming this highly lethal disease in children and in adults. In recent years, numerous review papers have been written outlining the conventional strategies in treating gliomas as well as those that have shown early in vitro or pre-clinical promise. Current strategies in overcoming the BBB for drug delivery, such as focused ultrasound and convection-enhanced delivery, as well as approaches involving nanoscale applications including liposomes, micelles, hydrogels and radiosensitisers, have previously been described in detail [9–11]. This review focuses on some of the more unconventional strategies that have had limited exposure but hold promise and potential in therapeutics targeting both pediatric and adult gliomas, from a directed perspective.

**Fig. 2:**
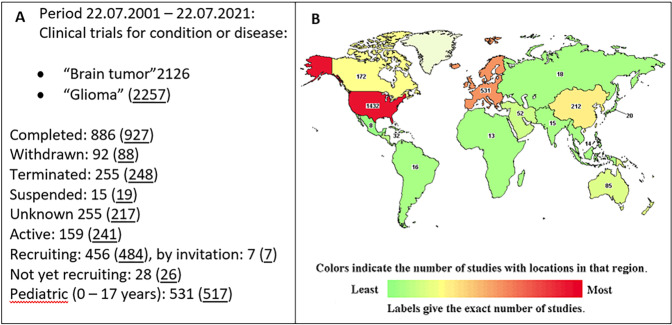
Distribution of clinical trials with access status and geographical region for brain tumor/glioma studies initiated over the last two decades. **a** Clinical trials, based on condition or disease, conducted in the 20-year period between 22.07.2001 and 22.07.2021, including status; **b**: World map of clinical trials by country. Source: ClinicalTrials.gov.

Although studies on both pediatric and adult gliomas are reviewed, most studies cited here are based on in vitro and pre-clinical adult glioma models, due to the lower availability of research material and a lack of literature in the pediatric field. In addition, enrolment of children in clinical trials is difficult, due to both low disease prevalence and parental reluctance, as well as lack of funding [12]. Nevertheless, registered clinical trials are steadily on the rise. Figure 2 outlines clinical trials registered on the ClinicalTrials.gov database between 22.07.2001 and 22.07.2021, based on condition or disease, of which just under 25% are pediatric (≤17 years).

## Cell-penetrating peptides

A major challenge in glioma therapy is the selective delivery of therapeutics to tumour cells behind the BBB, while limiting damage to other tissues. Although cell-penetrating peptides (CPPs) have been studied for nearly half a century, only the last decade or so has investigated their potential as a promising new strategy for targeted glioma treatment. CPPs are short peptides that can be coupled to various carriers for the selective delivery of drugs, imaging agents, nanoparticles, oligonucleotides and liposomes [13]. CPPs can target receptors which are overexpressed or limited to tumour cells as well as target abnormal signaling pathways. Through binding to their targets, these peptides can exert various effects, including the delivery of drugs. Although antibodies are often used in the clinic as tumour-targeting ligands, they nevertheless possess many limitations, including low stability in vivo, slow diffusion into the tumour tissue and high production costs. Compared to antibodies, tumour-targeting peptides have better BBB-penetrating capacities, traverse the cell membrane more readily and are easier to produce and modify [14]. Incorporating CPPs into drug delivery systems could therefore lead to more targeted and efficient glioma treatment.

Pre-clinical research has already shown promising results for CPP-mediated drug delivery in various cancer models, including glioma. A study in which the glioma homing CPP interleukin-13 peptide was anchored onto nanoparticles showed specific targeting and uptake by U87 MG cells, as well as enhanced penetration of particles into tumours both in vitro and in vivo [15]. When loaded with the chemotherapeutic docetaxel, these particles were also able to inhibit the growth of subcutaneous U87 MG tumours. In a similar study, conjugation of the glioma-targeting ligand transferrin with the CPP octa-arginine (R8) enhanced uptake and antiproliferative effects in U87 MG and GL261 glioma cell lines [16]. Another study showed a significant reduction of proliferation, migration and invasion of U87 MG cells in vitro following the uptake of a multifunctional construct consisting of the glioma homing peptide GL1, the tumour protein 53 (P53) C-terminal lysine-rich regulatory domain and the CPP polyarginine [17]. Similarly, it was shown that with small interfering RNAs a significant increase in gene-silencing specifically in U87 MG cells and not in non-glioma cells can be achieved by combining the targeting peptide angiopep-2 and the PepFect peptide 14 as a delivery system [18].

Nanocarriers conjugating CPPs and polyethylene glycol (PEG)-lipid conjugates have also been designed to enhance the targetability of tumours through prolonged circulation time and exposure at the tumour site [19]. The high expression of matrix metalloprotease 2 (MMP2) in the tumour microenvironment was utilised to cleave the PEG chains specifically at the target location, exposing the CPP, in turn promoting the internalisation of the nanocarriers into the tumour cells. A gene delivery system based on the cleavage of PEG-lipid micelles within the MMP2-rich tumour microenvironment was recently used to effectively deliver the DNA repair inhibitor DNA strand break bait (Dbait) into glioma by targeting the low-density lipoprotein receptor-related protein 1 (LPR1), which is implicated in BBB permeability while also being highly expressed in glioma [20]. Angiopep-2, which binds to LPR1, was conjugated to MMP2-responsive peptides, and used to deliver the Dbait payload, which also acts as a radiosensitiser, specifically to glioma cells in vitro and in vivo via the CPP octa-arginine, enhancing the effects of radiotherapy.

Although several brain-penetrating CPPs such as HIV-1 Tat, penetratin and SynB1 have been established and are being investigated to date, their number is still limited. A recent study showed that conjugating an 11 amino acid long CPP derived from the cell membrane entry signal of HIV-1 Tat to a chemically modified peptide mimicking H3K36me2 was able to inhibit the proliferation of DIPG cells harbouring a H3K27M mutation in vitro, while wild type cells were not affected [21]. As the potential of CPPs is becoming more evident, screens to identify novel and specific BBB-penetrating CPPs are being developed and are already showing results. Cho and colleagues developed a BBB-spheroid model for predicting BBB penetration in vitro. By screening a panel of 16 CPPs, the authors were able to show the utility of their system by identifying 4 potential CPPs in vitro and recapitulating their findings in vivo. Confocal microscopy and MALDI mass spectrometry revealed accumulation of all 4 CPPs within the brain parenchyma [22].

## Magnetic drug delivery/SPIO nanoparticles

Another emerging strategy to enhance the delivery of therapeutics across the BBB is magnetic drug delivery, in which external magnetic fields guide magnetic drug carriers towards the tumour site and increase local therapeutic efficacy. Superparamagnetic iron oxide nanoparticles (SPIONs), consisting of ﻿magnetite (Fe3O4) or maghemite (γ-Fe2O3), are promising magnetic carriers for this purpose, being biocompatible, biodegradable and integrated into the body’s iron cycle after degradation [23]. Surface modifications of SPIONs can also increase their biocompatibility and water dispersion properties, extend their blood circulation time by limiting nonspecific phagocytosis and provide active sites for drug loading [24]. The inherent magnetic properties of SPIONs also make them a promising option for both site-specific magnetic targeting and as contrast agents for ﻿magnetic resonance imaging (SPIO-MRI). To date, many SPIONs have been constructed as nanocarriers for glioma chemotherapeutics (for review see ref. [23]), with several clinical trials investigating their potential use in a variety of conditions, including brain neoplasms. Examples of current clinical trials utilising magnetic iron oxide nanoparticles (and the other strategies under review) are included in Table 1. Promising recent pre-clinical findings for the application of SPIONs as nanocarriers for magnetic chemotherapeutic drug delivery to glioma will be discussed below.

**Table 1:** Examples of clinical trials incorporating the strategies being reviewed.

ClinicalTrials.gov identifier	Year	Strategy	Phase	Sample size	Age (years)	Therapeutic applications	Endpoints	Status
NCT01975116	2013	Cell-penetrating peptides	I	18	3–21	CNS tumours	Side-effects, dosage	Completed, no results
NCT00914914	2009	Cell-penetrating peptides	I	15	≥18	Refractory solid tumour	Safety	Completed, no results
NCT00769093	2008	Iron oxide nanoparticles	I	6	≥18	HGG	Observe microvascular changes	Terminated^a^
NCT03179449	2017	Iron oxide nanoparticles	I	10	≥2	Malignant brain tumour	Macrophage characterisation	Recruiting
NCT03465618	2018	Carbon dots	I	10	≥18	HGG	Tumour localisation/distribution	Recruiting
NCT01266096	2010	Carbon dots	N/A	23	≥18	Malignant brain tumour	Characterisation	Active, not recruiting
NCT04015700	2019	Neoantigens	I	12	≥18	GBM	Safety and feasibility	Recruiting
NCT03914768	2019	Neoantigens	I	10	1–75	DIPG and GBM	Safety and potential benefit	Enrolling by invitation
NCT04749641	2021	Neoantigens	I	30	≥5	DIPG	Safety and preliminary efficacy	Recruiting
NCT04196413	2019	Neoantigens	I	54	2–30	H3.3K27M^+^ Glioma	Feasibility, dosage	Recruiting
NCT05038150	2021	Genetically modified bacteria	I	50	18–75	Refractory solid tumour	Safety and tolerability	Not yet recruiting
NCT02718443	2016	Attenuated bacteria	I	14	≥18	rGBM	Safety and tolerability	Completed
NCT03750071	2018	Attenuated bacteria	I	30	≥18	rGBM	Efficacy and safety evaluation	Recruiting

Source: ClinicalTrials.gov.

*CNS* central nervous system, *HGG* high-grade glioma, *GBM* glioblastoma multiforme, *DIPG* diffuse intrinsic pontine glioma, *rGBM* recurrent GBM, *N/A* not applicable.

^a^Inadequate enrolment.

Paclitaxel (PTX)-loaded SPIONs were recently established to assess the anti-tumour effects in human U251 GBM cells [25]. Uptake of PTX-SPIONs led to decreased cell proliferation and migration, and induced apoptosis. A comparable construct was prepared for an animal study, in which an external magnet was placed over the head of GBM-bearing mice [26]. Compared to controls, magnetic targeting increased the accumulation of PTX-SPIONs in the brain ex vivo and significantly prolonged median survival in vivo.

Temozolomide (TMZ)-loaded SPIONs conjugated to the glioma-targeting ligand folic acid (FA) have also been established for dual targeting of rat brain glioma [27]. In vitro assays showed that cytotoxicity of TMZ-SPIONs and TMZ-SPIONs-FA was 1.6 and 3.29-fold higher than free TMZ on rat C6 glioma cells, respectively. Analysis of MRI images before and after in vivo injections of TMZ-SPION-FAs showed an increased influx of particles into the brain in the presence of an external magnetic field, indicating successful crossing of the BBB. To evaluate the biodistribution of the nanoparticles with and without FA, the heads of rats were placed in a magnetic field after injection with particles. The distribution of nanoparticles in the vital organs far from the field was significantly reduced, and a comparison of brain and tumour tissues showed that nanoparticles containing FA had a significantly higher distribution in tumour tissue. The therapeutic efficacy of this dual method was demonstrated by prolonged survival time and reduced tumour volume of C6 glioma-bearing rats.

Doxorubicin (DOX)-loaded SPIONs (DOX-SPIONs) have also been developed to study magnetic drug delivery to glioma cells both in vitro and in vivo [28, 29]. The uptake of DOX-SPIONs by C6 cells under a magnetic field was significantly increased compared to free DOX in vitro, leading to increased cytotoxicity. In glioma-bearing rats, ex vivo assays demonstrated that the DOX-SPIONs were delivered to the tumours by imposing external magnetic fields. Furthermore, this treatment led to the complete suppression of glioma growth after 28 days in vivo. The safety of these particles was verified through histological analysis of major organs. Similarly, a co-culture model was also used to examine the BBB permeability and anti-tumour activity of DOX-SPIONs compared to free DOX [29]. While DOX-SPIONs demonstrated enhanced permeability, cytotoxicity was similar in both studies. However, the combination of a cadherin binding peptide to temporarily open tight junctions with an external magnetic field significantly improved both DOX-SPION permeability and cytotoxicity. As SPIONs clearly show potential, a follow-up study was performed in which a similar carrier loaded with ﻿salinomycin was established [30]. Consistently, results suggest that SPIONs combined with penetration enhancers and external magnetic fields could potentially lead to effective and specific magnetic targeting for glioma chemotherapy.

The potential of magnetic nanoparticles to target GBM cells ﻿that have spread within cerebrospinal fluid has also been explored [31]. Magnetic nanoparticles consisting of gold-iron cores bound to the chemotherapeutic agent etoposide, recognised for its intrathecal use, were established. These particles were shown to move at human size-distances in response to a rotating magnetic field. After remote targeting at 15 cm from the magnet, the etoposide particles significantly eradicated human U138 GBM cells compared to control particles in vitro. While further testing is necessary, intrathecal administration of magnetic drug carriers holds promise for the removal of glioma cells disseminated within the cerebrospinal fluid.

An alternative application of SPIONs is their utilisation as an imaging tool for delineation of resectable glioma in the operating room to guide surgeons. The use of fluorescent SPIONs for intraoperative imaging of GBM by targeting tumour-associated macrophages (TAMs) has already been investigated [32]. The fluorescent SPIONs selectively visualised TAM populations by in vitro live-cell imaging and in vivo fluorescence imaging. In orthotopic GBM xenograft models, the SPIONs could penetrate the BBB and successfully delineate the tumour. This study indicates the potential of SPIONs for improved intraoperative staging and more radical surgery.

## Carbon (nitride quantum) dots

Metal-free carbon nitride quantum dots (CDs) are nanoparticles with potential for biomedical applications as they harbour favourable properties such as good biocompatibility, photostability and tunable ﻿photoluminescence, as well as nontoxicity. Furthermore, being easy to produce and liquid dispersible makes them ideal candidates for application in bioimaging, drug delivery and theranostics [33]. The structural characteristics of CDs can also be modified for use in different biomedical applications. As CDs have a high loading capacity for chemotherapeutics, they are considered promising nanocarrier materials for cancer therapy. Non-selective interactions of some CDs with both tumour cells and normal cells have been previously reported and were seen as a disadvantage [34]. However, recent advances in CDs applications justify their investigation into glioma therapy strategies.

Using a simple one-step method, Ruan and colleagues prepared fluorescent carbon dots with demonstrated serum stability and low cytotoxicity, which could also be taken up by C6 glioma cells in vitro in a time- and concentration-dependent manner. In vivo, these carbon dots accumulated and distributed throughout the glioma in orthotopic C6 glioma-bearing mice, suggesting their potential in non-invasive glioma imaging applications [35]. Zheng and colleagues also prepared a fluorescent carbon dot variant, termed CD-Asp, based on D-glucose and L-aspartic acid, which exhibited excellent biocompatibility and a tunable emission spectrum. These carbon dots were seen to be highly selective for glioma cells in vitro, while in vivo analysis of C6 glioma-bearing mice showed that DC-Asp readily traversed the BBB and precisely targeted glioma tissue, with lower fluorescent signals being detected in normal brain tissue [36]. Both studies accentuate the potential of CDs in diagnostic, targeting and therapeutic functions, warranting further pre-clinical investigation.

Conjugates of CDs with the chemotherapeutic gemcitabine were investigated in their ability to selectively target and kill pediatric GBM cells in vitro [37]. Although gemcitabine has previously been shown to have potent anti-glioma effects in pre-clinical studies, human clinical trials have been largely disappointing [38], probably due to gemcitabine’s short plasma half-life, poor BBB penetration and dose-limiting toxicities. Conjugation of gemcitabine-CDs to transferrin showed the highly selective destruction of SJGBM2 glioma cells over non-cancerous HEK293 cells in vitro. BBB penetration capacity of these CDs was also demonstrated in vivo using a zebrafish model [37], highlighting the potential of CDs as nanocarriers for selective brain tumour drug delivery.

The synergistic effects of a triple conjugate targeting system, consisting of CDs conjugated to transferrin and the chemotherapeutic compounds epiburicin and TMZ, on pediatric and adult glioma cells, have also been investigated in vitro [39]. The efficacy of the triple system (two drugs and transferrin) was compared to dual systems (one drug and transferrin), ﻿non-transferrin CDs and free drugs. Results showed that the transferrin conjugated samples were more cytotoxic to the glioma cell lines compared to non-transferrin conjugates and that the triple systems were more cytotoxic than the dual systems. These results suggest that a triple conjugated CD system is a more capable therapeutic agent than single drug delivery systems.

Recently, a novel strategy to ﻿selectively image and deliver drugs to glioma using CDs structurally modified to mimic large amino acids that could bind to the large neutral amino acid transporter 1, which is expressed in most tumours, was examined [34]. As the BBB mostly limits drug delivery to brain tumours via traditional ligand-mediated methods, it was postulated that ﻿carrier transporters differentially upregulated in tumour cells might be a more effective approach. Results showed that intravenous injection of the CDs mimicking large amino acids selectively accumulated in an orthotopic mouse model of human glioma. Additionally, when loaded with the chemotherapeutic topotecan, these CDs enabled successful imaging of the tumours as well as significantly reduced tumour size without detectable toxicity.

A recent in vitro study combined the use of transferrin decorated magnetic nanoparticles with a different type of non-toxic quantum dots, namely ﻿InP/ZnS quantum dots, to investigate dual targeting and imaging of glioma [40]. The results demonstrated enhanced cellular uptake of these combined particles by glioma cells under an external magnetic field and showed both successful MRI imaging and improved fluorescence microscopy intensity. This is the first study to combine magnetic nanoparticles and quantum dots for anti-glioma therapy. However, further in vivo studies are needed to assess the safety and efficacy of this strategy in treating gliomas.

## Neoantigens and tumour-associated antigens

Neoantigens, or tumour-specific mutations, are more frequently being investigated as targets for glioma treatment utilising the immune response against the tumour. Advancements in cancer immunogenomics have allowed for the identification of targetable neoantigens, offering promise for the effectiveness of immunotherapy and priming it against tumours of the brain [41].

In comparison to adult brain cancer, pediatric brain cancers have less mutational load and corresponding neoantigens, and a tumour microenvironment with a smaller number of immune cell infiltrates. Consequently, immunotherapies developed for adult brain tumours are usually not as effective when applied to pediatric brain tumours, resulting in relatively fewer trials on immunotherapy for childhood glioma [42]. Fortunately, recent innovations have led to novel neoantigens and tumour-associated antigen strategies to be currently evaluated for adult as well as pediatric brain cancer.

A relatively recent neoantigen, ﻿lysine (K) to methionine (M) substitution at position 27 of histone 3 variant 3 (H3.3K27M), for glioma T cell therapy has been investigated rigorously since being identified [43]. ﻿The majority of pediatric diffuse midline gliomas (DMG) and more than 80% of DIPGs harbour this mutation, causing a global reduction of methylation on H3K27 and aberrant transcription of oncogenes. An HLA-A*02.01^+^ CD8^+^ T cell clone was established through stimulation with a synthetic peptide containing the H3.3K27M mutation. These T cells effectively killed HLA-A*02.01^+^H3.3K27M^+^ glioma cells in an antigen- and HLA-specific manner in vitro. Transfer of these T cells also efficiently inhibited the progression of glioma xenografts in mice.

Recently, ﻿the safety and efficacy of a H3.3K27M-targeted peptide vaccine were assessed in the Pacific Pediatric Neuro-Oncology Consortium [44]. Newly diagnosed patients, with HLA-A*02.01^+^ and H3.3K27M^+^ status, were enrolled in a DIPG (stratum A; 19 patients) or non-pontine DMG (stratum B; 10 patients) arm of the trial. The vaccine was administered together with an immunostimulant every 3 weeks for 8 cycles, followed by once every 6 weeks. Overall, the vaccine was well-tolerated and patients with ﻿H3.3K27M-specific CD8^+^ immunological responses showed prolonged overall survival compared to non-responders (16.1 vs. 9.8 months, *p* = 0.05). Although no significant differences in overall survival between the two strata were observed, these results warrant further studies targeting the H3.3K27M epitope.

The disialoganglioside GD2 is another tumour-associated antigen that has gained interest in cancer immunotherapeutics due to its limited expression in normal human tissues [45] and uniform, high expression in patient-derived H3.3K27M^+^ glioma cell cultures [46] and in glioblastoma [47]. A first-in-human trial was initiated in 2019 to determine the feasibility of manufacturing autologous GD2 expressing CAR-T cells, assess their safety and determine the maximum tolerated dose in children and young adults with H3.3K27M^+^ diffuse gliomas (ClinicalTrials.gov Identifier: NCT04196413). Results from the first four patients enrolled in the study were recently published, and show that the treatment is well-tolerated and that 3 of 4 patients derived clinical and radiographic benefits following intravenous administration of GD2-CAR-T cells. A second dose of GD2-CAR-T cells via intracerebroventricular administration provided additional radiographic/clinical benefits in three of three patients treated. Additionally, on-target, off-tumour toxicity was not observed in any of the patients. Although it is early in the trial, with only a few patients tested, the current results of this therapeutic approach are promising and encouraging for patients of glioma [48].

Tumour-associated antigens or neoantigens enable selective targeting (e.g. through immunotherapy) of the glioma microenvironment. However, such targets may be expressed on some tumour cells but not on others, which can result in some cells escaping immunotherapy. To limit tumour immune evasion, patients should receive immunotherapy that targets multiple neoantigens. For example, a recent study developed a novel multivalent vector protein, QUAD 3.0, which concurrently targets four GBM associated Eph receptors [49]. This approach allows the entire tumour microenvironment to be covered while minimising the chance of antigen loss and reducing therapeutic resistance. Additionally, QUAD 3.0 conjugated to DOX was shown to be cytotoxic to both established and patient-derived GBM cell lines in vitro, with IC_50_ values in the low nanomolar range. Given the positive results observed so far, multivalent vector proteins could be further used in pre-clinical models in the foreseeable future. Although immunotherapeutic intervention should be regarded with caution, the development of immunotherapies focusing on recruitment, activation and retention for pediatric glioma is warranted to harness the potential of this strategy in treating brain tumours in children.

## Tumour-targeting bacteria

Recent developments in targeted therapy and immunotherapy have raised hopes for the treatment of glioma. However, some challenges concerning these approaches remain to be addressed, including toxicity to healthy tissues, the inability to treat deep tumour tissue and the possibility of inducing drug resistance in tumour cells [50, 51].

Although not a novel concept, live tumour-targeting bacteria offer a unique opportunity to meet these challenges. Compared to other strategies, tumour-targeting bacteria have multiple capabilities for suppressing cancer [50]. These bacteria preferably accumulate and proliferate within tumours, where they can trigger anticancer immune responses. For example, anaerobic bacteria specifically colonise the uniquely hypoxic tumour microenvironment and locally exert oncolytic effects. Bacteria can be modified via simple genetic manipulation or synthetic bioengineering to produce and deliver anti-tumour agents. Live tumour-targeting bacteria can be used either as a monotherapy or combined with other anticancer therapies to optimise clinical outcomes. The mechanisms of selective induction of tumour regression, different engineering methods and clinical trials involving tumour-targeting bacteria are reviewed elsewhere [51]. Here we describe recent advances in using live tumour-targeting bacteria for anti-glioma therapy.

It has previously been demonstrated that the attenuated tumour-targeting and highly mobile *Salmonella typhimurium* (S. *typhimurium*) ﻿A1-R strain can suppress and eliminate human glioma in an orthotopic mouse model [52]. ﻿S. *typhimurium* A1R is auxotrophic (leu ⁄arg-dependent) but receives sufficient nutrition from tumour tissue and does not continuously infect normal tissue. In one study, glioma-bearing mice were treated with S. *typhimurium* by intravenous or intracranial injection once a week for three weeks and observed via intravital microscopy. Intracranially administered bacteria were shown to inhibit brain tumour growth at 7.6-fold higher compared to untreated mice and significantly improved survival, with two of ten mice appearing to have their tumours eliminated. The same study showed that intravenous administration of bacteria was not effective. Another study used S. *typhimurium* A1-R to assess its anti-tumour efficacy through intravenous or intrathecal injection in orthotopic mouse models of spinal cord glioma [53]. Intrathecal treatment was seen to significantly improve survival and inhibited hind-limb paralysis compared to the intravenously treated and control groups.

*Salmonella species pluralis* are facultative anaerobes which can survive in oxygenated environments [51]. Through improved tumour targeting, reduced toxicity or increased efficacy of this bacteria could potentially be achieved. Since numerous cancer types, including glioma, overexpress the αvβ3 integrin, an S. *Typhimurium* strain displaying an integrin-binding Arg–Gly–Asp(RGD) peptide on its outer membrane to enhance specific targeting was designed [54]. This strain showed a > 1000-fold enrichment in U87 MG xenografts in mice compared to control. In a similar study, a different type of enhanced tumour-targeting S. *typhimurium* bacteria was designed [55]. This strain had mutations in the *﻿msbB* gene, blocking systemic toxicity, and in the ﻿*purI* gene, rendering it incapable of synthesising purines. Since purines are highly detectable in tumours but barely detectable in healthy tissues, these mutations generated a tumour seeking tendency that could be exploited. These bacterial carriers could induce apoptosis via a combination of the expression of tumour suppressor protein P53 and the pro-apoptotic drug Azurin. In a xenograft model of human U87 MG GBM in rats, intracranial injection of bacteria significantly prolonged survival. Histological and proteomic analyses showed no systemic toxicity and a restored neural environment in treated responders.

*Clostridium novyi* (*C. novyi*), an avirulent, highly mobile anaerobic bacteria that is extremely attracted to the hypoxic tumour microenvironment was attenuated and shown to induce an effective anti-tumour response in an orthotopic rat F98 glioma model [56]. The tumours were engineered to express luciferase, and a rapid fall of luciferase activity was observed within 48 h after intratumoral *C. novyi* spore injection, indicating a reduction in tumour volume. *C. novyi* specifically localised to the tumour, sparing surrounding normal cells and were observed to eliminate islands of microinvasive tumour cells that had diffused within healthy brain tissue. This bacterial treatment also resulted in a significant survival advantage. Abscess formation was not observed in this model with the adequate use of antibiotics. However, abscess formation in the brain remains a potential risk of *C. novyi* therapy in humans and would require excision and drainage. Given the poor prognosis of gliomas, the benefits of *C. novyi* therapy might compensate for these potential side-effects. This study also had encouraging results in a canine model, which ultimately led to treating a human patient. In a comparable study, intravenously injected spores of *C. novyi* led to tumour destruction and significantly prolonged survival in both rat F98 and human 060919 brainstem glioma xenograft models in rats [57]. As with the previous study, *C. novyi* germination was specific to the tumour, with sparing of the healthy brain parenchyma. However, oedema and increased intracranial pressure could have been fatal if not properly managed with hydration and antibiotics.

## Conclusions

While this review is by no means exhaustive in describing the treatment strategies currently under investigation for treating high-grade gliomas, the aim is to bring to light some of the atypical recent advancements and stratagems that hold potential in advancing glioma treatments in children and adults. Gliomas present an unmet medical need and have done so since being described. The challenges that these tumours present can be seen in the radical strategies being attempted to improve prognosis, and eventually find a curative treatment for this disease. Although the ultimate goal is to defeat glioma, this victory must not be Pyrrhic, with considerations for quality of life intertwined with treatment strategies. Delivery systems and combined modality treatment strategies need to be investigated thoroughly, with events such as neurotoxicity and additive effects of treatment elucidated in efficient experimental models so that a therapeutic index with a favourable benefit-risk ratio can be achieved. Table 1 shows a concise list of clinical trials incorporating the strategies being reviewed for brain neoplasms. Table 2 lists the strategies discussed above and their investigation over the last two decades, including studies and clinical trials that have been initiated and registered on the clinicaltrials.gov server [58], including those that are glioma specific. With advancements in modern medicine, immunotherapy targeting neoantigens could hold the key to finding a cure for gliomas, which is evident through the number of clinical trials being initiated. Nevertheless, sometimes we must look back and revisit what we may have missed to have a clearer view of the future. Over 90 years ago the neurologist Foster Kennedy stated “He who cares for patients suffering from brain tumour must bring to his problem much thought and stout action. There is also a need for formidable optimism, for the dice of God are loaded”. This statement still holds true today, with optimism being resolute in the vigorous search for the cure of gliomas.

**Table 2:** Publications and clinical trials involving the five strategies discussed above in the last 20 years up to and including 22.07.2021.

Strategy [2001–2021]	Published articles		Clinical trials	
	[22.07 2001–22.07 2021]			
		Glioma specific		Glioma specific
Cell-penetrating peptides	4716	122	2	1
SPIONs	13,362	302	19	2
Carbon (nitride quantum) dots	180	3	6	2
Neoantigens (TAAs)	2400 (9848)	58 (258)	176 (500)	13 (39)
Tumour-targeting bacteria	21,400	502	68	6

*TAAs* tumour-associated antigens.

## Supplementary information

Declaration of competing interests
